# Supplementary material for: Deep history of cultural and linguistic evolution among Central African hunter-gatherers
Source: Nat Hum Behav. 2024 May 27;8(7):1263–75. doi: 10.1038/s41562-024-01891-y (PMC11272592; doi:10.1038/s41562-024-01891-y)
Supplement: Supplementary file 1 — Supplementary Texts 1 and 2, Figs. 1–21, Tables 1–22, and legends to Datasets 1 and 2. [file 41562_2024_1891_MOESM1_ESM.pdf]

nature human behaviour

Article https://doi.org/10.1038/s41562-024-01891-y

Deep history of cultural and linguistic evolution among Central African hunter-gatherers

In the format provided by the authors and unedited

This PDF file includes:

- Text S1to S2
- Figs. S1 to S21
- Tables S1 to S22
- Legends to Datasets S1 to S2

Other Supplementary Materials for this manuscript include the following:

- Dataset S1
- Dataset S2

Supplementary text 1: Extended explanation of fractioning of genetic sample and IBD analyses

After removing Bantu-associated genomic regions using the local ancestry inference and masking procedure described in Materials and Methods in the main text, we introduced a new procedure to split the remaining genetic data (associated exclusively with CAHG ancestry) into fractions representing distinct temporal depths.

IBD analysis allows the identification of genetic sequences that two individuals share due to a recent common ancestor (a recent relative). In other words, an IBD fragment represents a DNA sequence found in individuals 1 and 2, which is too similar (or too long), to have evolved independently. For this reason, most IBD fragments will be identified in individuals from the same population, as they are more likely to have a common recent ancestral relative. However, if the two individuals 1 and 2 were sampled from two distinct populations (for example, from two CAHG groups), they must also have inherited the similar sequence from a recent common progenitor. We can therefore infer that a recent migrant between the two populations introduced the sequence from one population into the other. Note that despite the misleading name, an IBD fragment found between two populations is identical by descent from a recent migrant or progenitor (a parent, in the case of siblings), not from a remote member of the ancestral population prior to the split between the two populations. Moreover, although IBD fragments between populations contributed to our measure of 'genetic distance' between CAHG populations (as they were included in some genetic sets), strictly speaking they only provide a measure of recent genetic flow or admixture. Hence, IBD analysis provides no information on the genetic distance between populations since their split, or divergence dates from an ancestral population. It only provides evidence for recent gene flow resulting in sequence similarities not compatible with observed levels of genetic differentiation between populations.

The other feature of IBD fragments is that they do not last indefinitely. Every generation, due to crossing-over and recombination, the chromosomes introduced by a migrant will incorporate fragments from the local background, and the size of sequences (IBD length) similar between the individual descending from the migrant in population 1, and individuals in the source population 2, continuously decreases. The longer the time since the migration event, the shorter the shared IBD fragments become, until it is no longer possible to tell that the sequence was derived from a recent migration event. For this reason, IBD analysis can only inform us of recent but not remote gene flow. However, this also means that the size of the IBD is inversely proportional to how long ago the migration/admixture event happened: a long shared IBD sequence means very recent migration, while smaller sequences mean a more remote migration event. In addition, a migration date also provides an estimated rate of migration: an identified migration 1,000 years ago suggests a rate of one migration event per 1,000 years, as the more recent event may have been preceded by others no longer identifiable.

In our study, we proposed an extension of the IBD technique. As discussed, we first excluded genomic regions of Bantu origin based on the masking procedure in the main text. Hence, the remaining distribution of genetic similarities reflected only genetic admixture between CAHG groups (before or after the Bantu arrival), but not between CAHG and Bantu groups. Finally, we also excluded IBD fragments exchanged between CAHG groups. What is left were short IBD fragments unlikely to represent recent migration, or corresponding to older migrations that cannot be dated. In parallel, we assessed shared musical instruments between the same CAHG populations, obtaining a measure of cultural similarity. We then tested for a correlation between cultural and genetic similarities. We ran the analyses for each set of genetic data: full set, set with CAHG-derived IBDs only (after masking of Bantu-derived ancestry fragments), and set excluding all recent IBD fragments. We repeated the procedure for cultural distances based on subsistence tools.

Supplementary text 2: Extended explanation of the assessment of the sharing of terms to design musical instruments and subsistence tools.

To judge whether words used to designate the same objects were sufficiently similar to suggest borrowing or common origins, for each word, first we checked whether our recorded terms appeared in the language descriptions of the corresponding group and that they had consistent meanings. When available, we worked with the assumption that diverging terms between pairs of languages could be partly explained by differences in the words' morphology. In the case of Bantu languages, a prevalent feature is the noun class prefix system which can differ between languages without impact on the similarity of the root. For example, the differences between the word for arrow used by the Bakoya (*di.banzika*[1]) and the Aka (*mbànzà*[2]) could be attributed to the fact the in these two languages the Bantu singular / plural noun class system differs (See Jacquot[3] for a description of the Bakoya noun class and Bouquiaux & Thomas[4] for that of the Aka). If the noun class prefixes are disregarded, similarities between the roots become more transparent (*banz*).

We also considered orthographic conventions that might have led to different representations of terms due to differences in linguists' backgrounds rather than real differences in the meaning or origins of the terms. For instance, Bantu voiceless alveolar affricates can be either transcribed as *ts* or as *tch* as a result of French influence, and homorganic nasals preceding labiodental fricatives can also vary in the way they are transcribed (e.g.: *mf/nf* or *mv/nv*)[5].

Importantly, surface differences between terms can also reflect regular phonological processes, either diachronic or synchronic, such as the vowel shifts between *sòkò* (Aka) and *sàkà* (Baka), or *lìgbégbé* (Baka) and *li.gbo:gbo* (Efe)[6]. Such differences point to a shared origin. When words are borrowed, however, phonological processes can also be idiosyncratic. As a general rule we posit such processes and therefore shared vocabulary only when the processes reflects a phonetically natural process or superficial variation. Transcriptions of tones are unreliable in many sources, making it impossible to establish similarity when tones differ. After compiling the final list of shared musical instrument and subsistence tool terminology between CAHG groups, we also noted whether the objects denoted by the terms were unique to CAHGs (as opposed to present in other African non-hunter-gatherer groups) as well as whether the terms themselves were unique to CAHGs (see Dataset S2 for notes). After doing so, we created two datasets of shared specialized vocabulary: one including all shared terms between CAHG groups, and one including only those terms that were unique to CAHG, and where evidence of common origin/ borrowing was clear.

References:

1. Soengas, B. La subsistance des Pygmées Bakoya à l'épreuve de l'agriculture: dynamique des savoirs ethnobotaniques et des pratiques (Département de la Zadié, Ogooué-Ivindo, Gabon). (Museum national d'histoire naturelle-MNHN PARIS, 2010).
2. Kilian-Hatz, C. *The Linguistic Link between (Western) baMbenga and (Eastern) baMbuti Pygmies*. (Academia-Verlag, 2019).
3. Jacquot, A. *Les classes nominales dans les langues bantoues des groupes B. 10, B. 20, B. 30 (Gabon-Congo)*. vol. 157 (IRD Editions, 1983).
4. Bouquiaux, L. & Thomas, J. M. Quelques problèmes comparatifs de langues bantoues C10 des confins oubanguiens: le cas du mbati, du ngando et de l'aka. *Sprachen und Sprachzeugnisse in Afrika: eine Sammlung philologischer Beiträge Wilhelm JG Möhlig zum* **60**, 87–106 (1994).
5. Schroeder, L. Bantu orthography manual. *E-book. SIL International. Retrieved March* **15**, 2015 (2008).
6. Nurse, D. Toward a typology of diachronic phonological change in Bantu languages. *Linguistica Atlantica* 100–122 (1987).

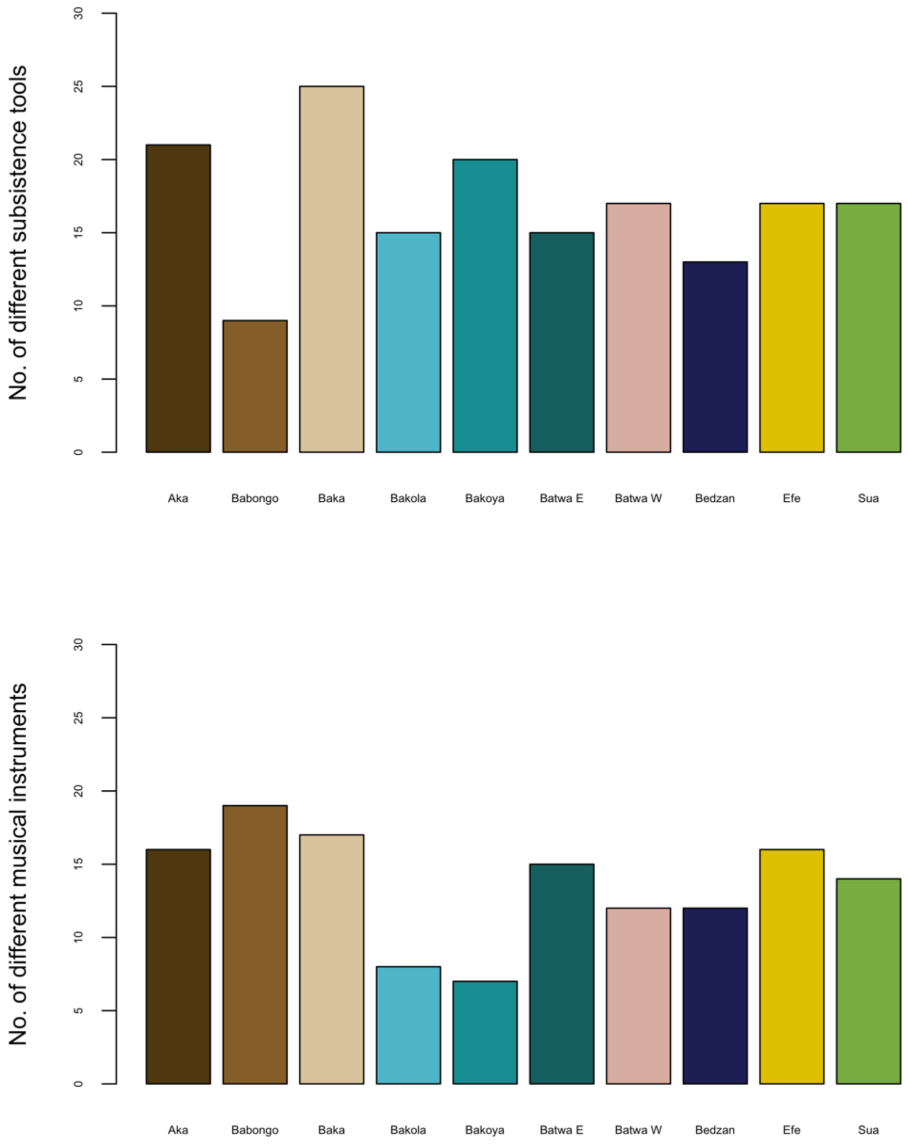

Fig S1. Count of different subsistence tools (top) and musical instruments (bottom) for the 10 CAHG populations in our sample.

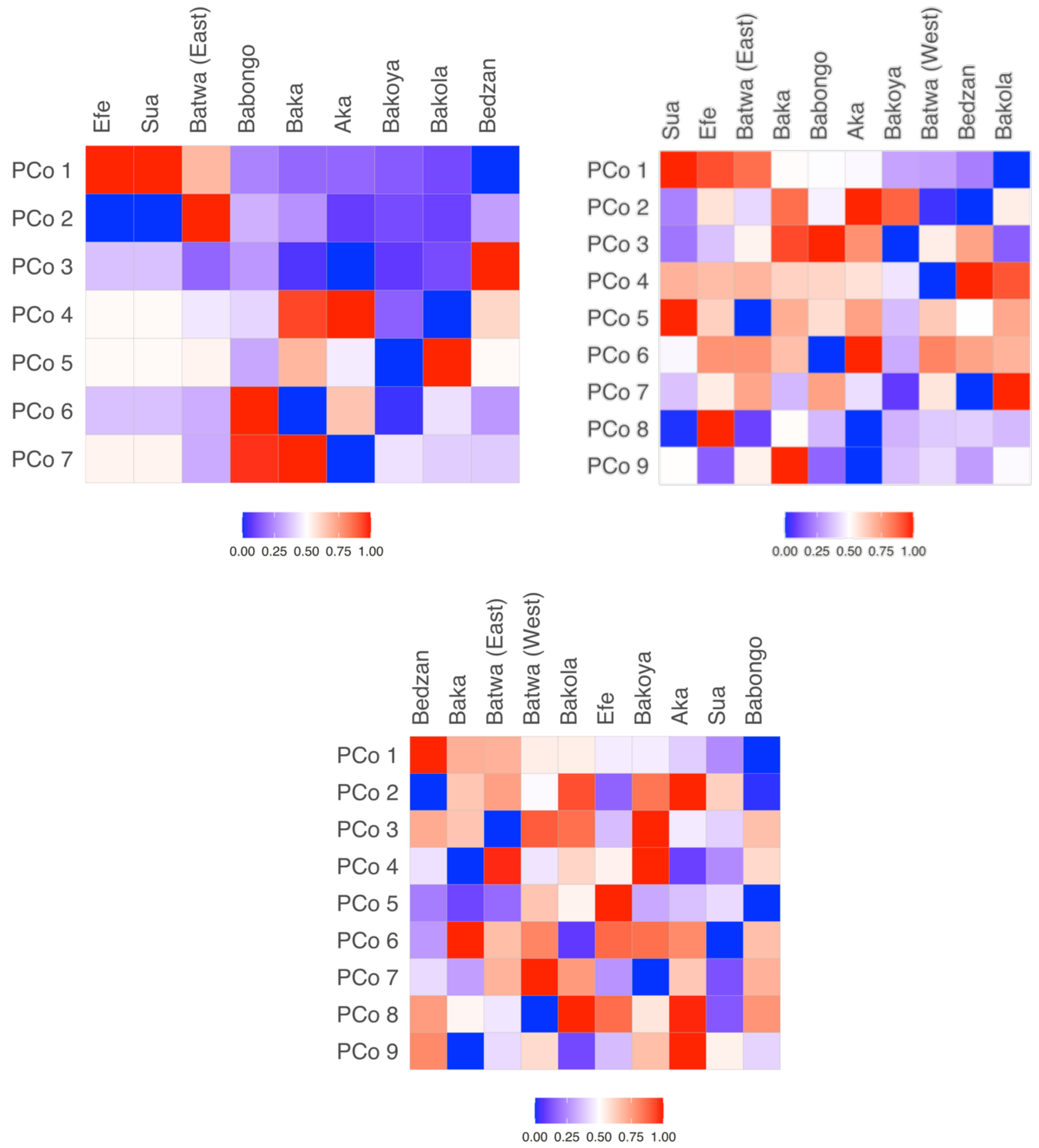

Fig S2. Contribution to PCoA heatmaps in genes using exclusively CAHG ancestry components (top left), music (top right) and subsistence toolkits (bottom)

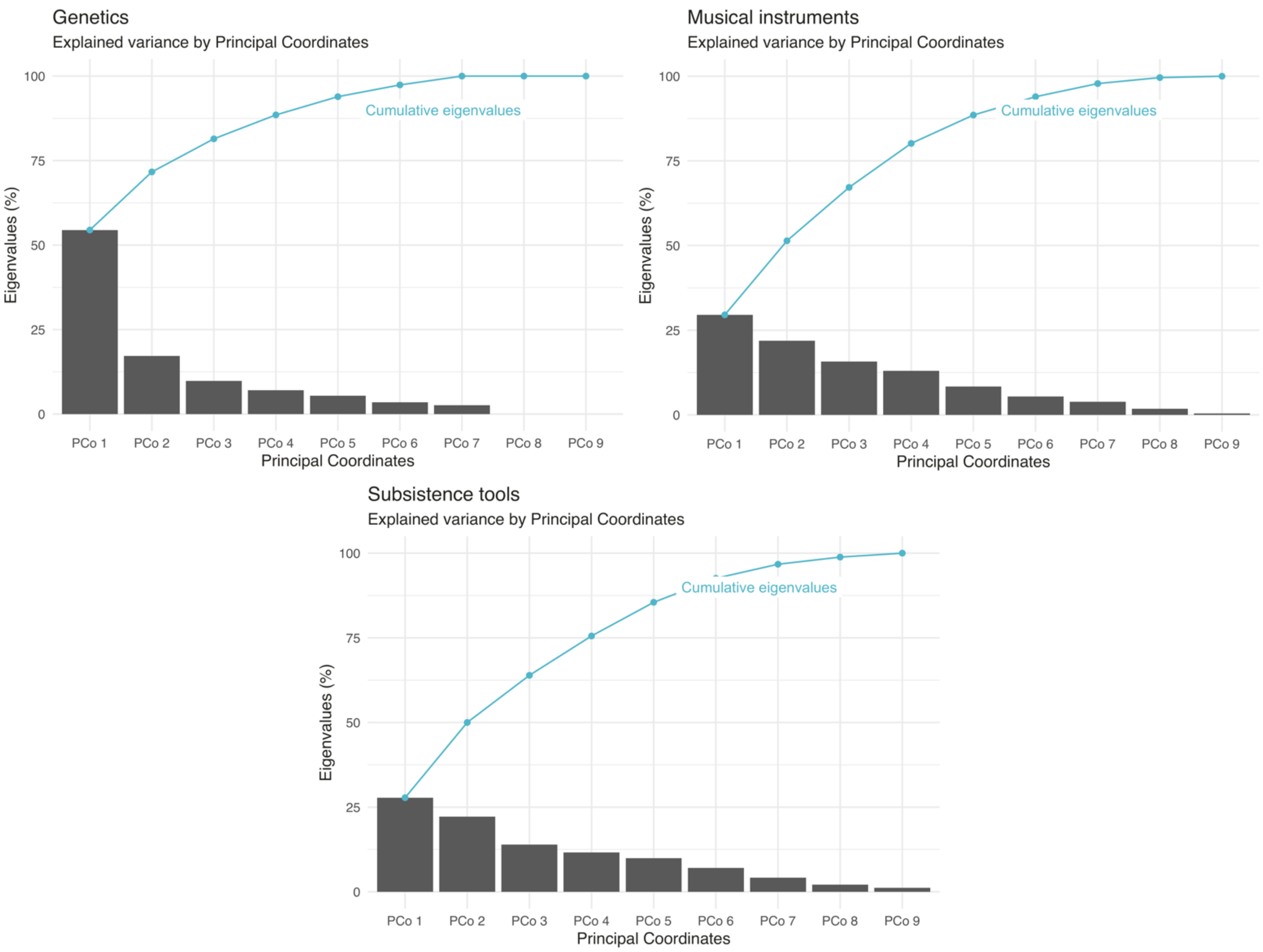

Fig S3. Explained variance by each principal coordinate in PCoA analyses for genes (top left), music (top right) and subsistence toolkits (bottom). Blue line indicates cumulative percentage of variance explained by adding each successive principal coordinate.

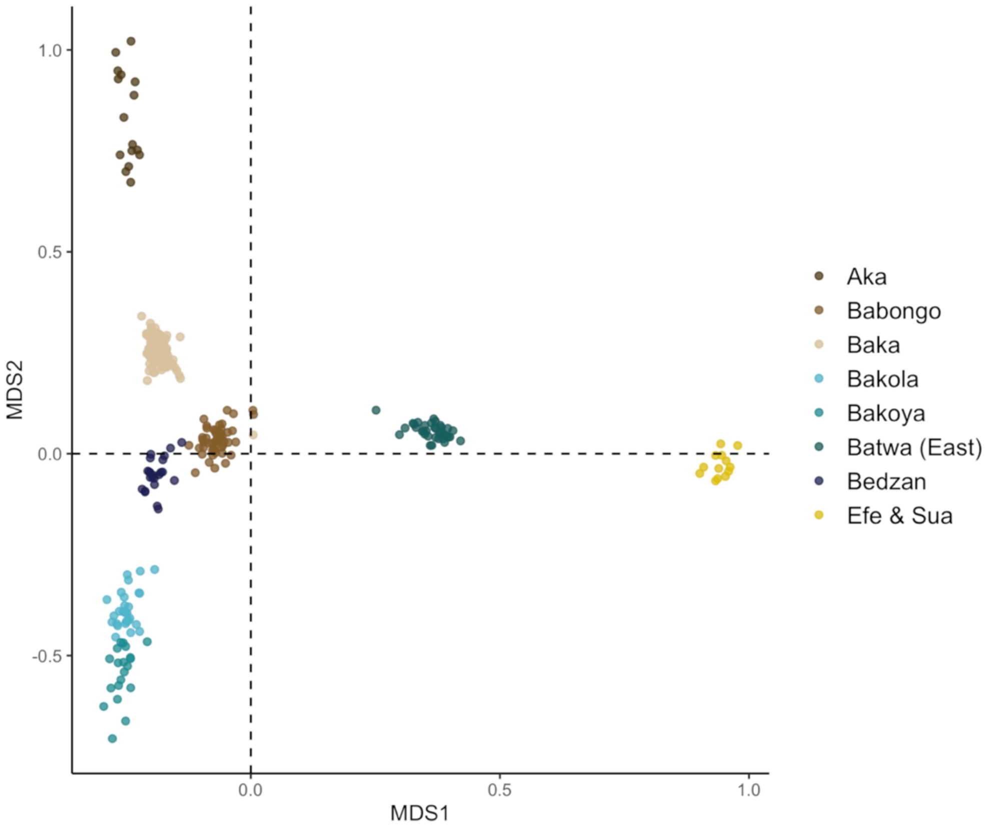

Fig S4. Multidimensional scaling (MDS) of CAHG ancestry components revealing segregation of Western and Eastern groups, and North-western and South-western groups.

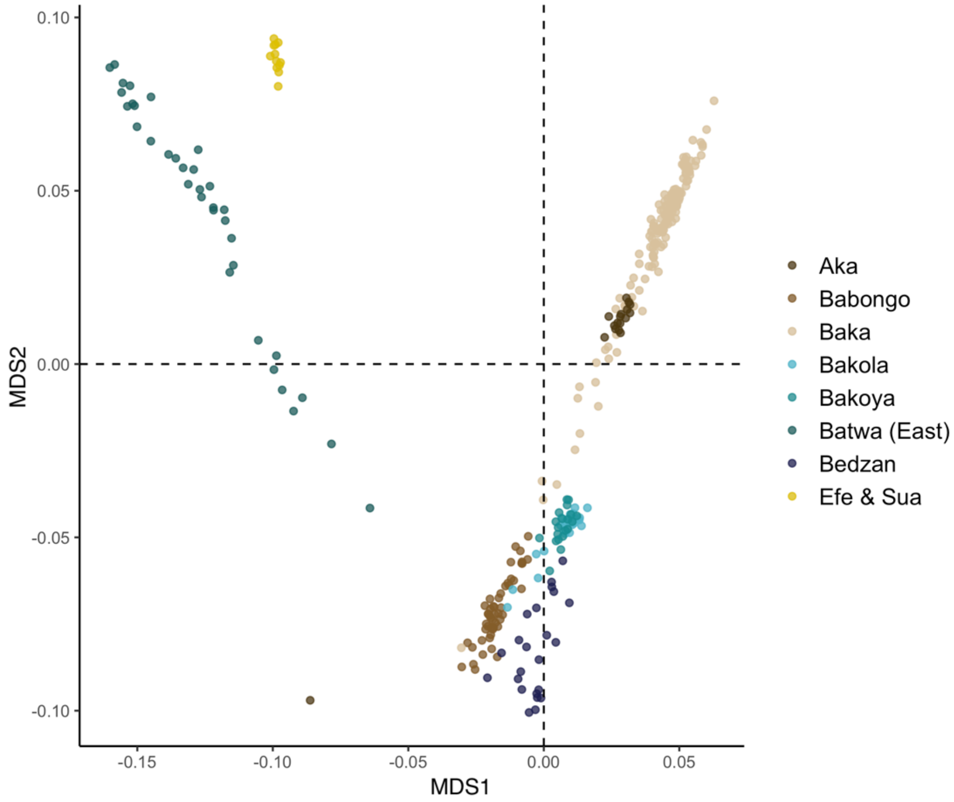

Fig S5. MDS of CAHG with available genome-wide SNP data using the full (unmasked) dataset

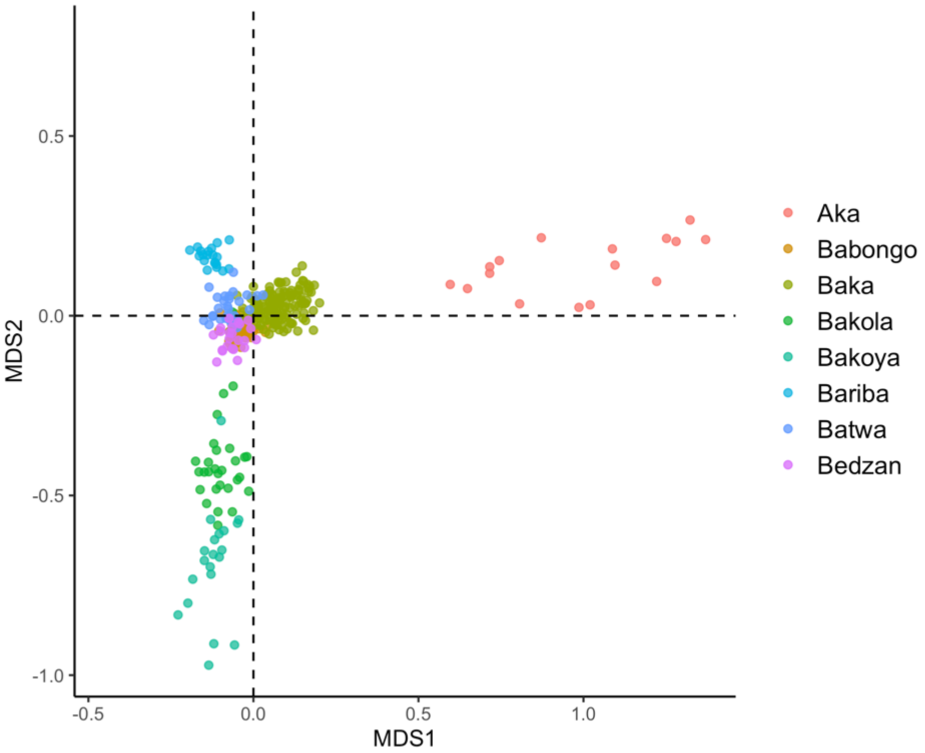

Fig S6. MDS of CAHG with available genome-wide SNP data using exclusively Bantu-associated genomic segments. Note that the Efe & Sua (Mbuti) are not included as they do not carry a sufficient amount of Bantu-associated DNA.

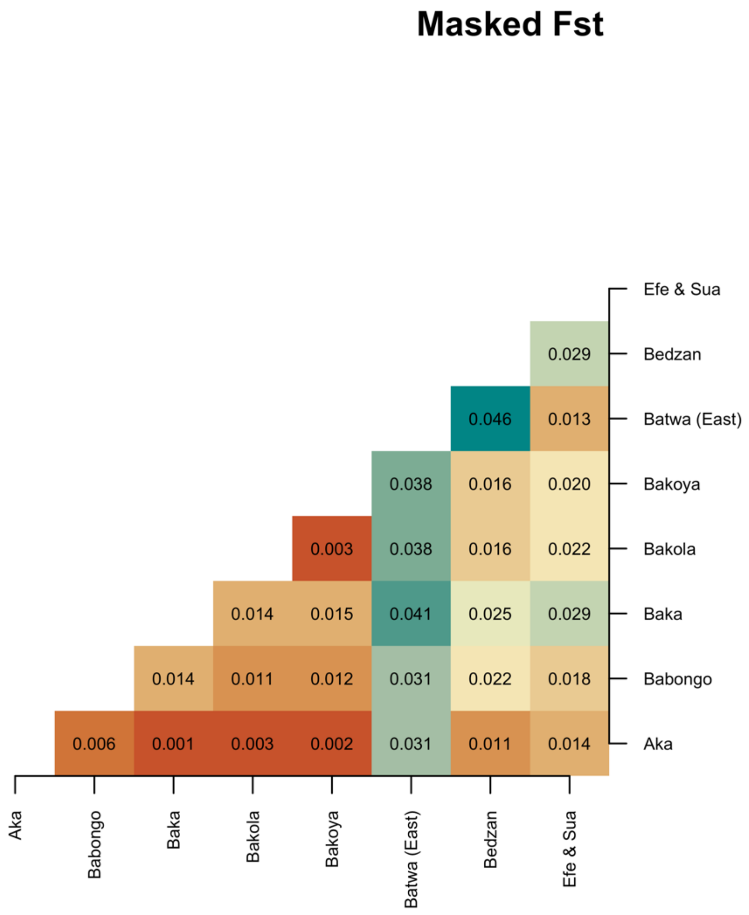

Fig S7. Inter-population F_{ST} using only CAHG ancestry components

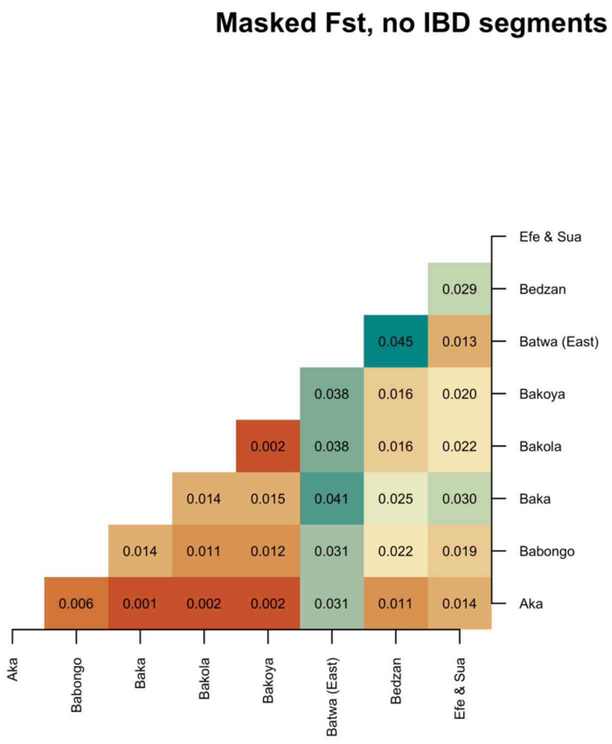

Fig S8. Pairwise F_{ST} matrix using only CAHG ancestry components and excluding shared IBD segments between populations

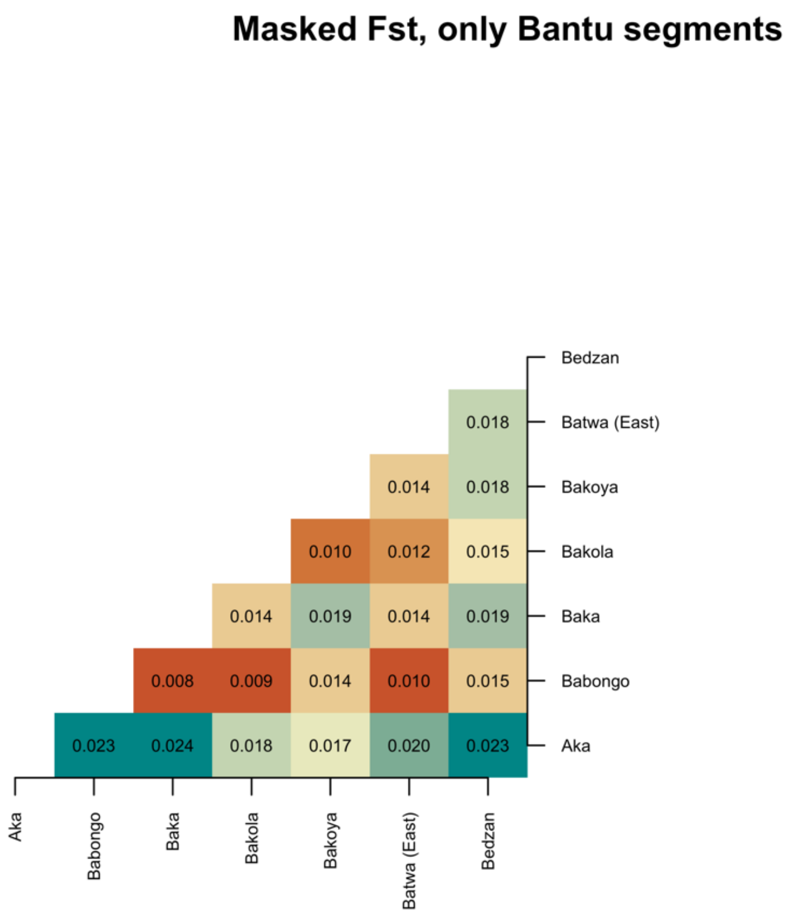

Fig S9. Pairwise F_{ST} matrix using only Bantu ancestry components.

Biomes

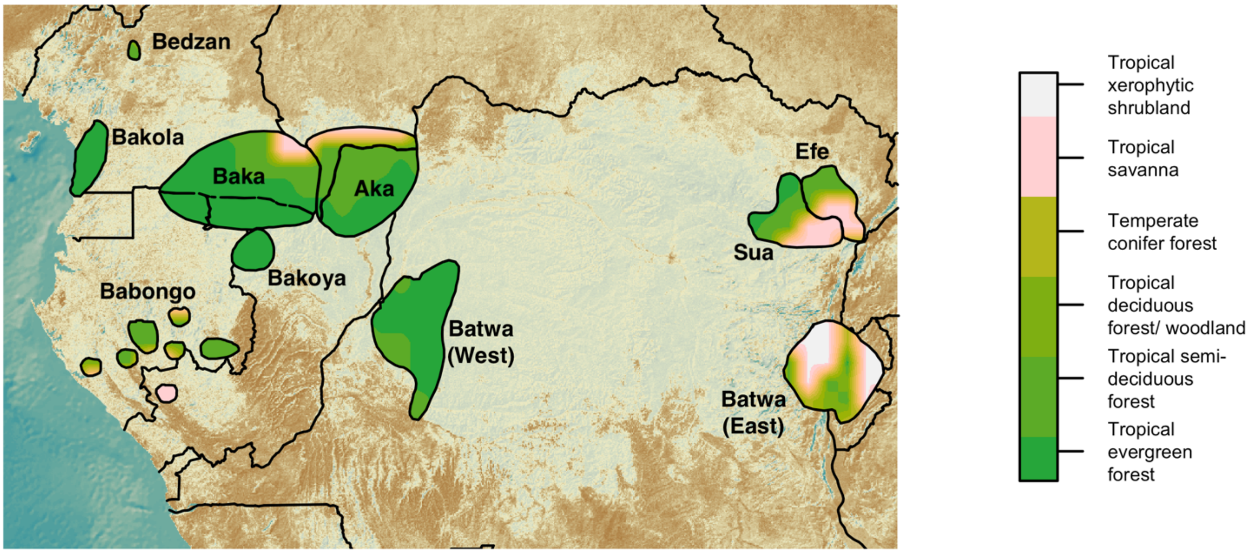

Fig S10. Biomes in the territories of the cultural groups included in our analyses obtained from Beyer et al. (2020)

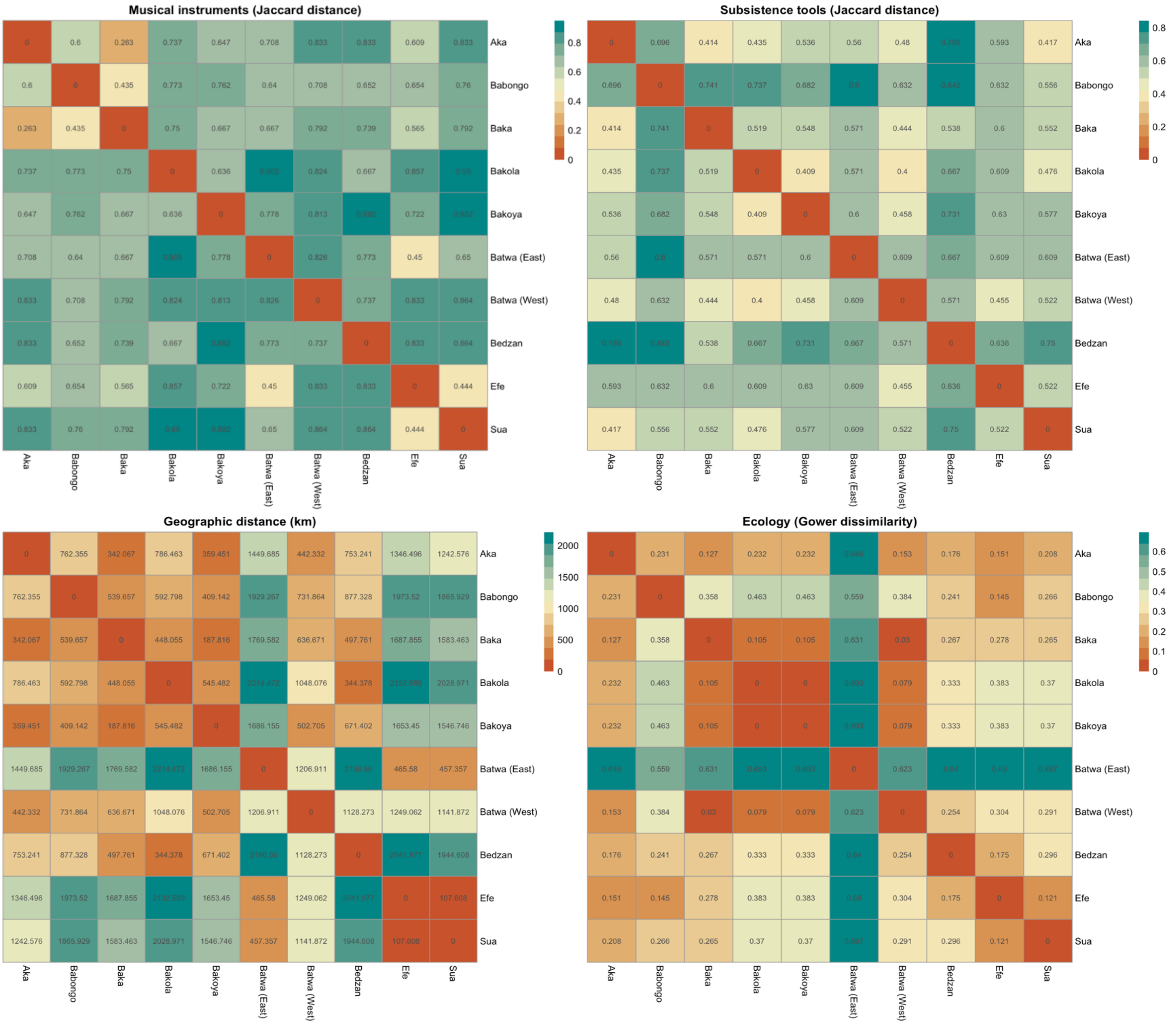

Fig S11. Between-population distance matrices used for the analyses.

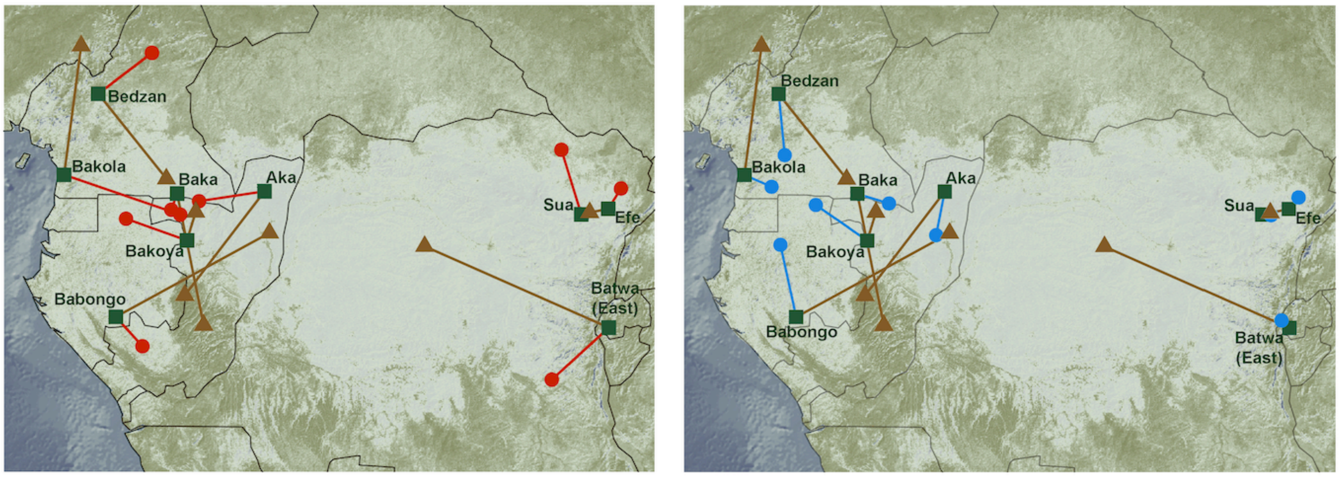

Fig S12. (Left) SpaceMix results showing pseudo-coordinates of CAHG groups based on subsistence tools (red dots), masked genomic data (triangles) and actual geographic coordinates (squares). (Right) SpaceMix results showing pseudo-coordinates of CAHG groups based on musical instruments repertoires (blue dots), masked genomic data (triangles) and actual geographic coordinates (squares).

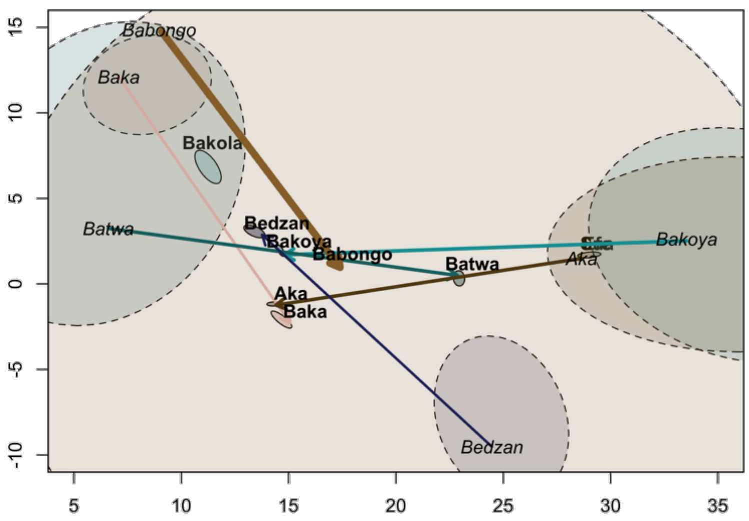

Fig S13. Estimated recent gene flow between CAHG by SpaceMix. Bold letters indicate geo-genetic coordinates of populations in our sample, Italic letters indicate sources of admixture, and arrows indicate direction of admixture. Ellipses bound by solid lines indicate 95% confidence interval around the estimated geo-genetic location of each population and ellipses bound by dashed lines 95% confidence intervals around the hypothesised admixture source. Width of arrows is proportional to estimated proportion of ancestry of a population deriving from admixture source location (origin of the arrows).

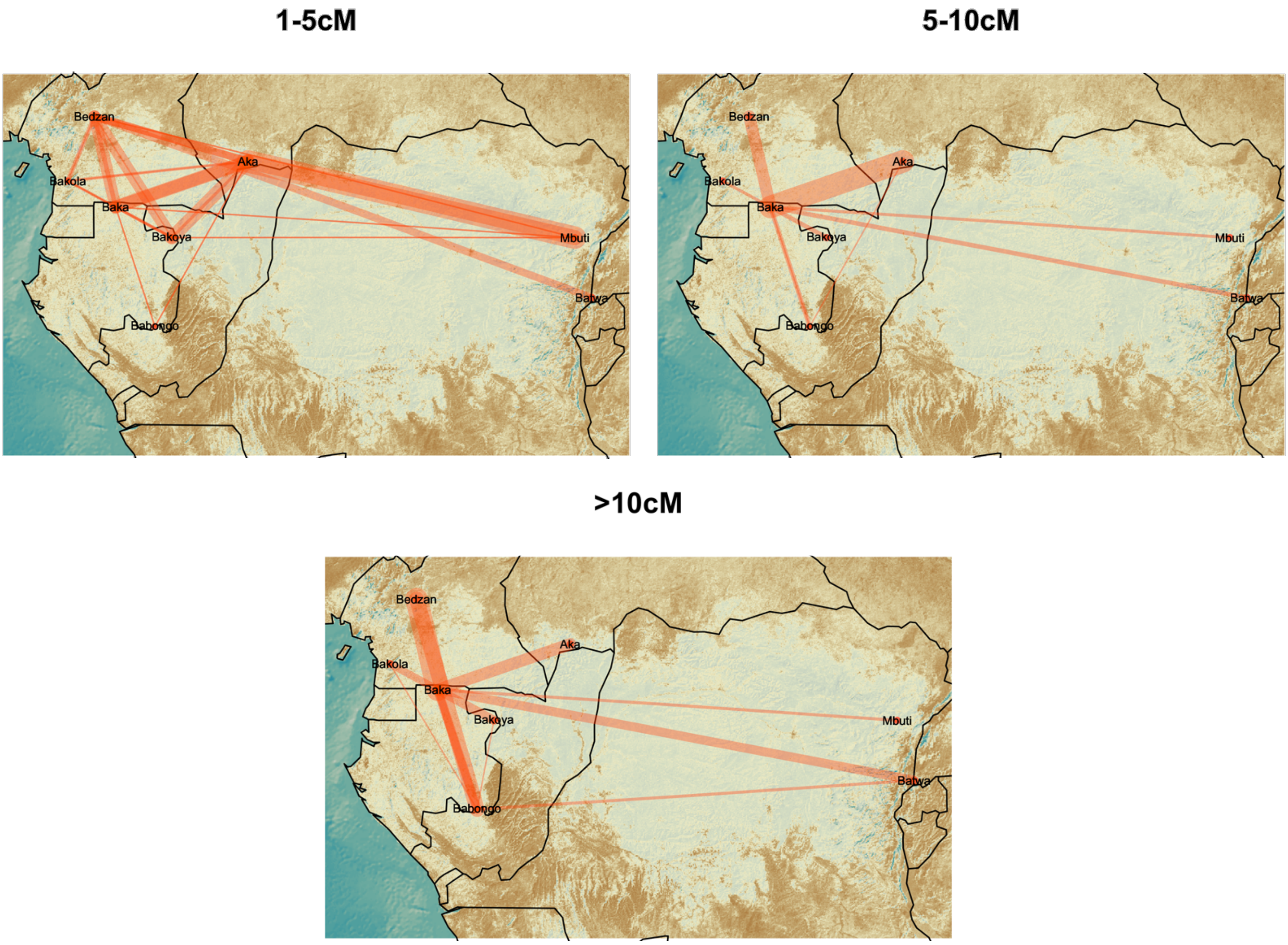

Fig S14. Recent genetic connectivity between CAHG populations. Network visualizations of the average number of IBD segments shared per cross-population individual pairs using only CAHG genetic segments with identified IBD blocks in the range of: (top-left) 1 to 5 cM (2,500 to 1,500 y ago), (top-right) 5 to 10 cM (1,500 to 500 y ago), (bottom) and over 10cM (500 to 0 y ago). Thicker lines indicate greater gene flow as identified by a higher probability of sharing IBD blocks of the specified length.

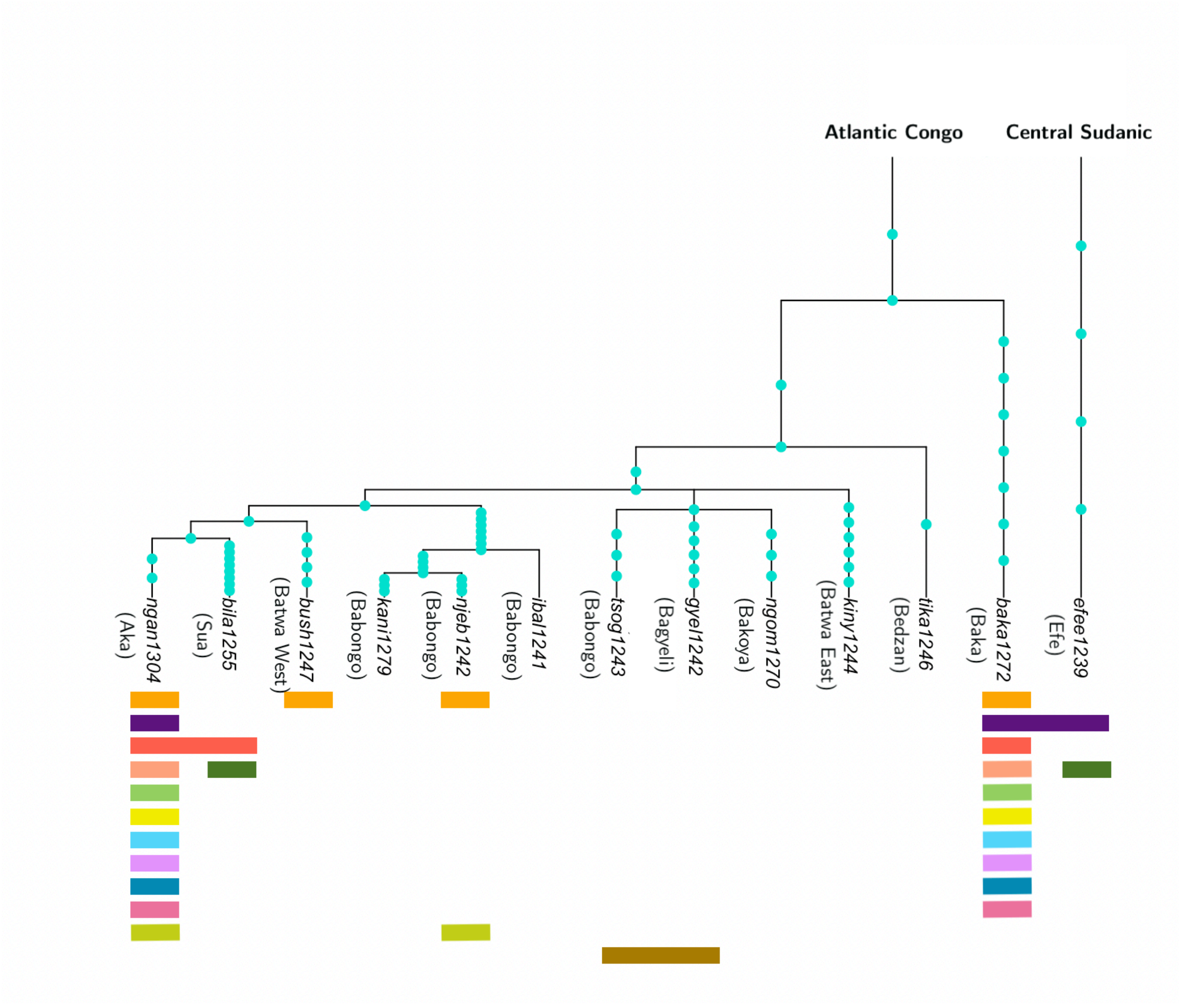

Fig S15. Glottolog tree including all languages spoken by the CAHG groups in this study. In italics, the Glottocodes of the languages in Table S3. The blue dots represent the internal nodes of the trees (Atlantic Congo and Central Sudanic) separating the languages. Each coloured rectangle present in multiple languages represents a musical instrument word shared between corresponding languages.

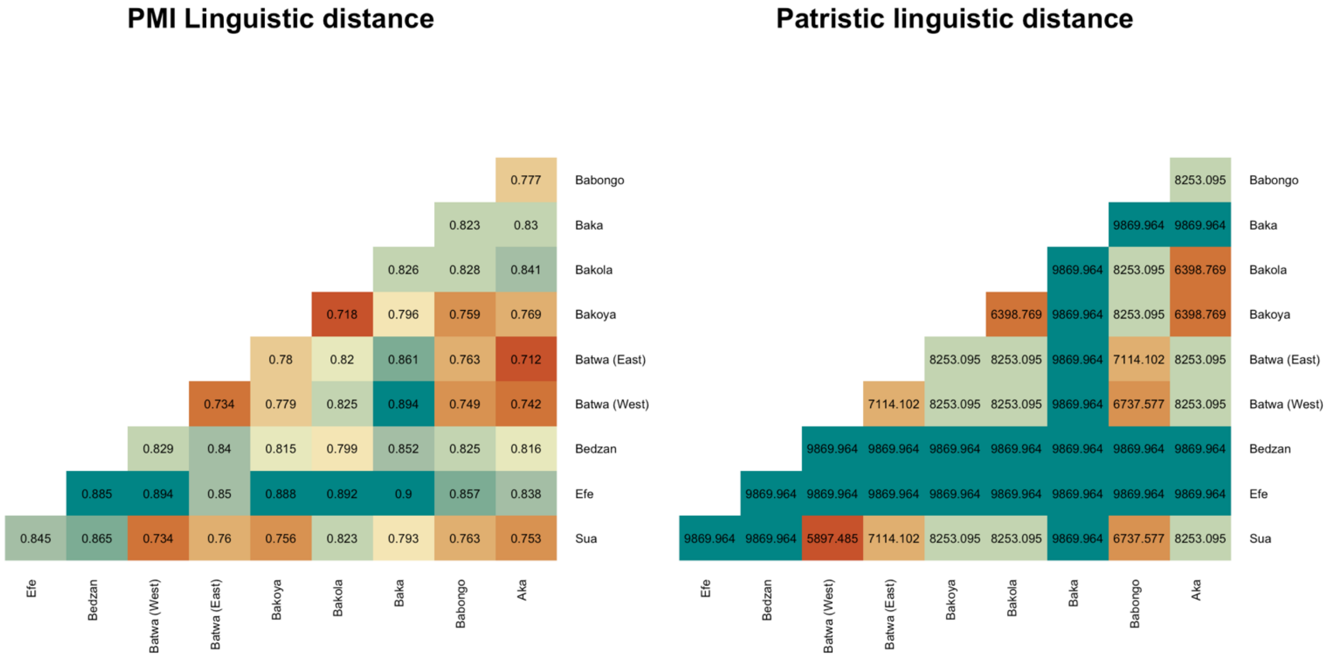

Fig S16. Between-population linguistic distance matrices averaged across all the languages spoken by each population based on Pointwise Mutual Information (PMI) distances (left) and patristic distances (right) (see Tables S2-S3). See *Materials and Methods* for detailed explanations on these measures. For non-Bantu speaking populations (Efe, Bedzan and Baka) a patristic distance equal to the maximum depth of the Bantu phylogenetic tree was used (9869.964).

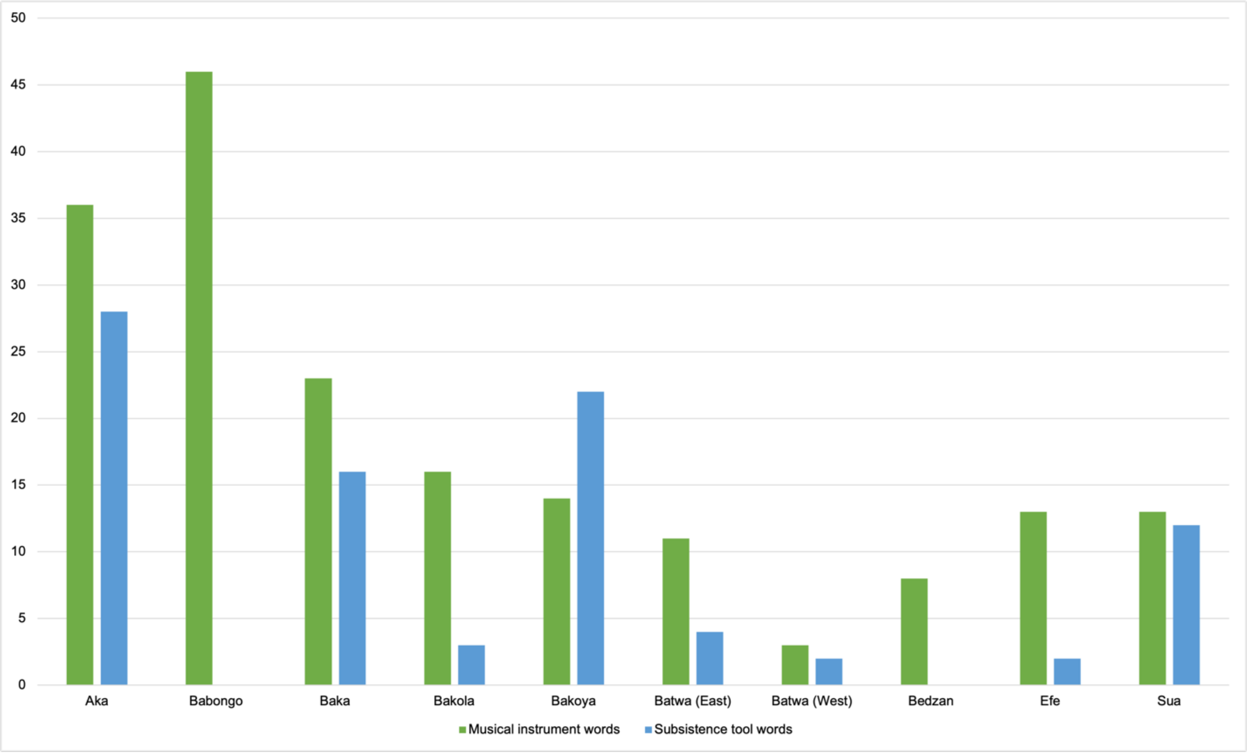

Fig S17. Number of different words compiled per population to designate musical instruments and subsistence tools.

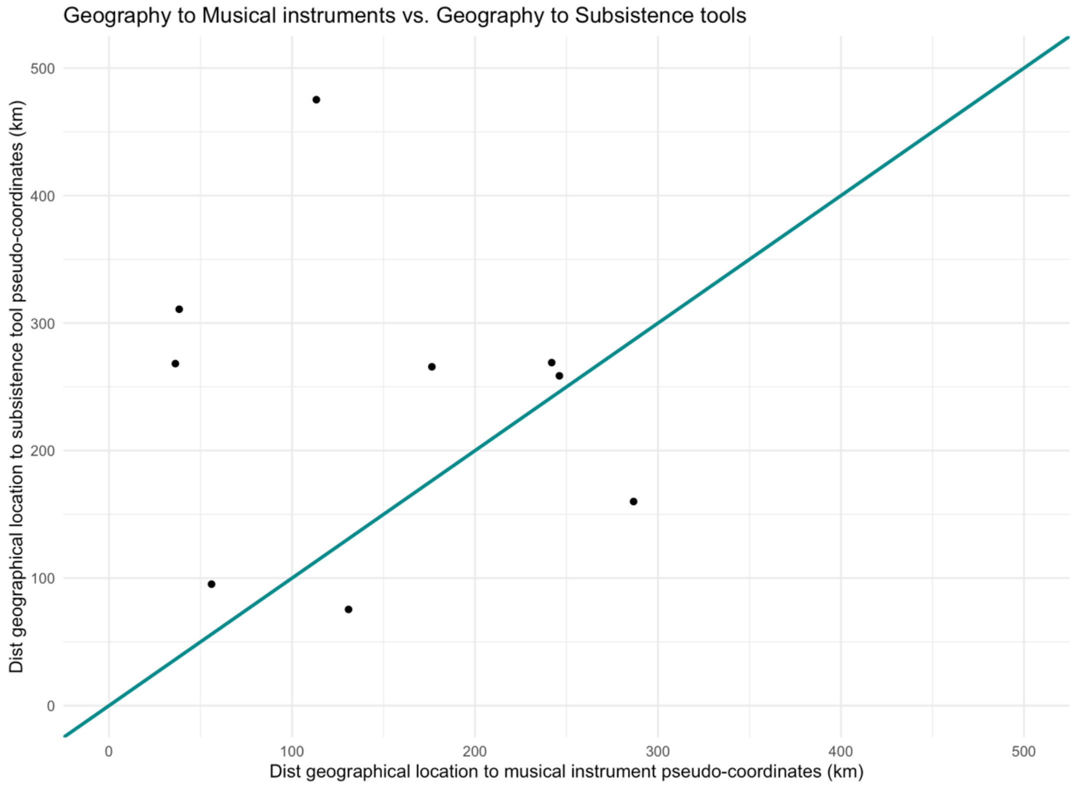

Fig S18. Comparison of distances between *SpaceMix* pseudo-coordinates based on musical instrument repertoires to the real geographical coordinates of CAHG populations against distance between pseudo-coordinates based on subsistence tool repertoires to the real geographical coordinates of CAHG populations. Blue line indicates where *y*=*x* (i.e. both distances are the same).

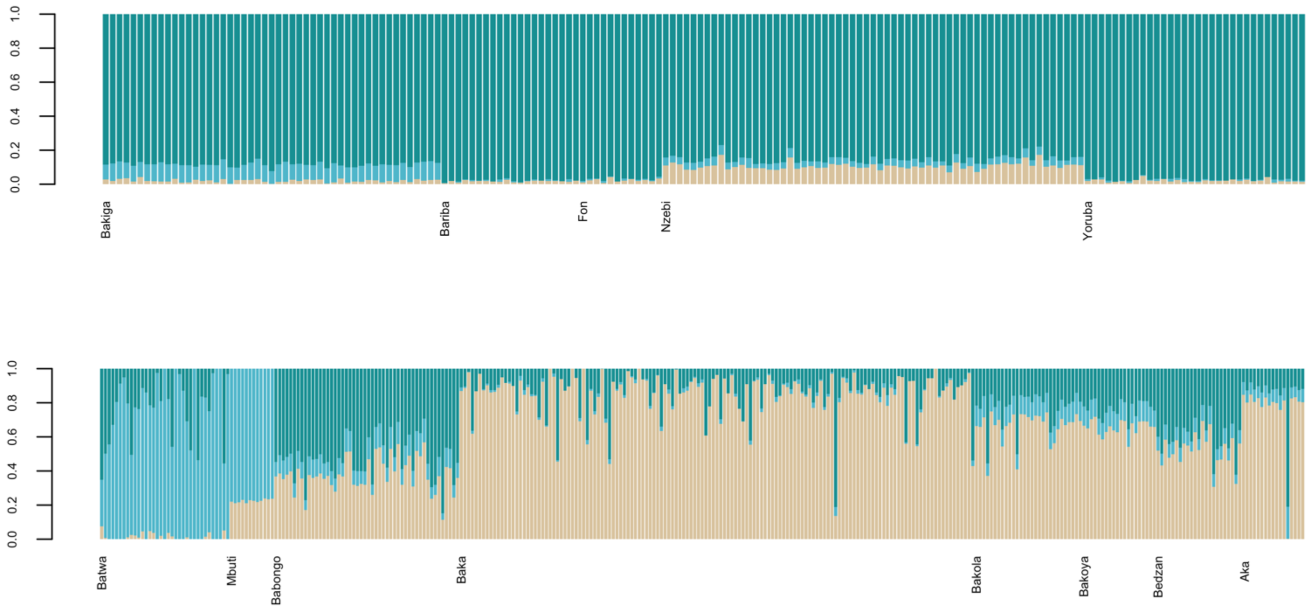

Fig S19. Results from the programme ADMIXTURE on our reduced pruned SNP dataset. Each bar corresponds to an individual and colours represent the proportion of inferred ancestry components from K=3 ancestral populations**.** Note the "Mbuti" group comprises the Efe and Sua.

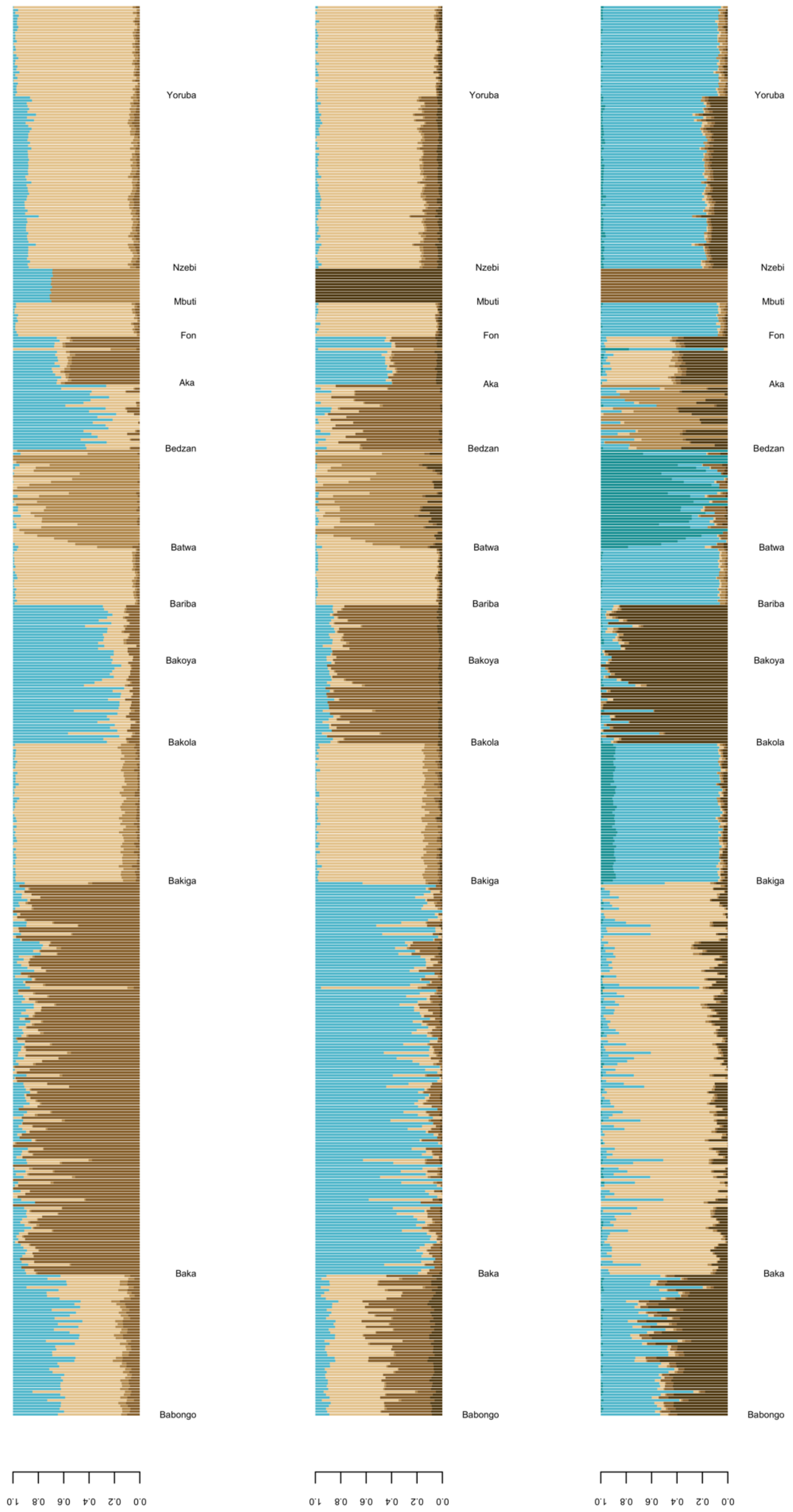

Fig S20. Admixture results at K=4 (left), K=5 (middle) and K=6 (right). Note the "Mbuti" group comprises the Efe and Sua.

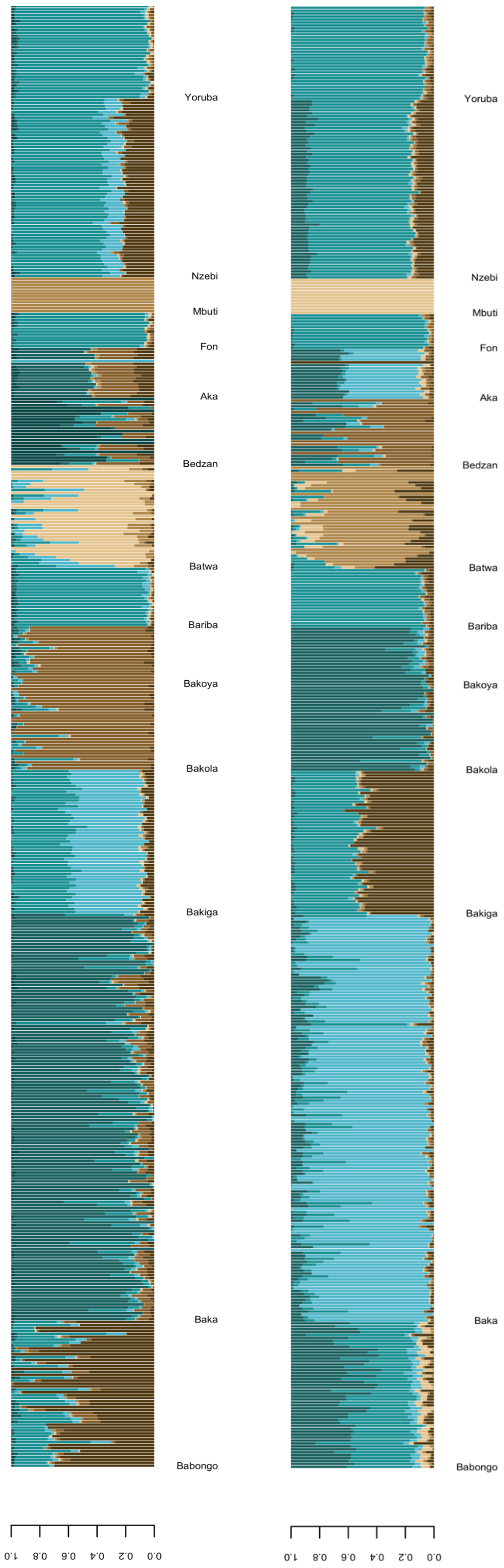

Fig S21. Admixture results at K=7 (left) and K=8 (right). Note the “Mbuti” group comprises the Efe and Sua.

Table S1. Area, and location of each group after Bahuchet 2006;2012 and estimated population size of each of the groups by Bahuchet 2006; Hewlett and Fancher, 2013 (western Batwa), and Lewis, 2000 (for the eastern Batwa). For Babongo, we merged his estimate for central and southern Babongo.

Culture	Lon	Lat	Area (degrees squared)	Estimated census pop size	Country
Aka	17.22	2.43	6.09	30,000-50,000	CAR, Congo
Babongo	11.98	-2.00	1.84	9,500	Gabon
Baka	14.14	2.35	8.01	30,000-40,000	Cameroon, Gabon, Congo
Bakola	10.16	3.01	1.12	4,000	Cameroon
Bakoya	14.49	0.70	0.88	2,600	Gabon, Congo
Batwa (East)	29.35	-2.38	4.45	70,000-87,000	DRC, Uganda, Rwanda
Batwa (West)	18.53	-1.33	4.89	14,000	DRC
Bedzan	11.36	5.87	0.11	400	Cameroon
Efe	29.33	1.81	1.73	10,000	DRC
Sua	28.38	1.62	2.44	26,000	DRC

Table S2. Linguistic classification of the languages spoken by the CAHG populations included in this study from Bahuchet 2006;2012 (Following classification by Greenberg, 1966; Ruhen, 1991 and Guthrie, 1967-71).

CAHG group	Phylum	Stock	Family	Group	Sub-group
Baka	Nigel-Kordofanian	Niger-Congo	North Central	Adamawa-Ubangian	Gbanzili-Sere
Bedzan	Nigel-Kordofanian	Niger-Congo	South Central	Bantoid, Non-Bantu	Tikar
Bakola	Nigel-Kordofanian	Niger-Congo	South Central	Northwest Bantu	A80
Bakoya	Nigel-Kordofanian	Niger-Congo	South Central	Northwest Bantu	B20
Babongo	Nigel-Kordofanian	Niger-Congo	South Central	Northwest Bantu	B30
Babongo	Nigel-Kordofanian	Niger-Congo	South Central	Northwest Bantu	B60
Babongo	Nigel-Kordofanian	Niger-Congo	South Central	Northwest Bantu	B70
Aka	Nigel-Kordofanian	Niger-Congo	South Central	Northwest Bantu	C10
Batwa (West)	Nigel-Kordofanian	Niger-Congo	South Central	Northwest Bantu	C60
Batwa (East)	Nigel-Kordofanian	Niger-Congo	South Central	Central Bantu	J11
Batwa (East)	Nigel-Kordofanian	Niger-Congo	South Central	Central Bantu	J60
Sua	Nigel-Kordofanian	Niger-Congo	South Central	Central Bantu	D30
Efe	Nilo-Saharan	Central-Sudanic	East Central	Mangbetu-Efe	-

Table S3. Linguistic classification of the languages spoken by the CAHG populations included in this study from the Ethnologue. For those CAHG languages have not been described or classified, we write the classification of the closest language (CL) from the closest farming language from which the CAHG language is considered a dialect of, after verifying that both were classified as belonging to the same linguistic family and subgroup according to Bahuhet, 2006.* Denotes groups that do not speak a language from the Bantu family.

CAHG group	Ethnologue entry (ISO 639-3 code)	Exact language/ closest neighbour	Glottocode	Ethnologue classification
Baka*	Baka (bkc)	EL	baka1272	Niger-Congo, Atlantic-Congo, Volta-Congo, North, Adamawa-Ubangi, Ubangi, Sere-Ngbaka-Mba, Ngbaka-Mba, Ngbaka, Western, Baka-Gundi
Bedzan*	Tikar (tik)	CL	tika1246	Niger-Congo, Atlantic-Congo, Volta-Congo, Benue-Congo, Bantoid, Southern, Tikar
Bakola	Gyele (gyi)	EL	gyel1242	Niger-Congo, Atlantic-Congo, Volta-Congo, Benue-Congo, Bantoid, Southern, Narrow Bantu, Northwest, A, Makaa-Njem (A.801)
Bakoya	Koya (nra)	EL	ngom1270	Niger-Congo›Atlantic-Congo›Volta-Congo›Benue-Congo›Bantoid›Southern›Narrow Bantu›Northwest›B›Kele (B.22)
Babongo	Kaningi (kzo)	EL	kani1279	Niger-Congo›Atlantic-Congo›Volta-Congo›Benue-Congo›Bantoid›Southern›Narrow Bantu›Northwest›B›Mbete (B.602)
Babongo	Teke (tek)	CL	ibal1241	Niger-Congo›Atlantic-Congo›Volta-Congo›Benue-Congo›Bantoid›Southern›Narrow Bantu›Northwest›B›Teke (B.75)
Babongo	Tsogho (tsv)	CL	tsog1243	Niger-Congo›Atlantic-Congo›Volta-Congo›Benue-Congo›Bantoid›Southern›Narrow Bantu›Northwest›B›Tsogo (B.31)
Babongo	Nzebi (nzb)	EL	njeb1242	Niger-Congo, Atlantic-Congo, Volta-Congo, Benue-Congo, Bantoid, Southern, Narrow Bantu, Northwest, B, Nzebi (B.52)
Aka	Ngando (ngd)	CL	ngan1304	Niger-Congo›Atlantic-Congo›Volta-Congo›Benue-Congo›Bantoid›Southern›Narrow Bantu›Northwest›C›Ngondi
Batwa (West)	Bushong (buf)	CL	bush1247	Niger-Congo, Atlantic-Congo, Volta-Congo, Benue-Congo, Bantoid, Southern, Narrow Bantu, Northwest, C, Bushoong (C.83)

Batwa (East)	Twa (kin)	EL	kiny1244	Niger-Congo, Atlantic-Congo, Volta-Congo, Benue-Congo, Bantoid, Southern, Narrow Bantu, Central, J, Ruanda-Rundi (D.61)
Sua	Bila (bip)	CL	bila1255	Niger-Congo, Atlantic-Congo, Volta-Congo, Benue-Congo, Bantoid, Southern, Narrow Bantu, Central, D, Bira-Nyali
Efe*	Efe (efe)	EL	efee1239	Nilo-Saharan›Satellite-Core›Satellites›Central Sudanic›East›Mangbutu-Efe

Table S4. Populations included in our genetic analyses.

Population	N	Name in original datasets	Meta group
Babongo	50	BabongoE/BabongoS	West CAHG
Baka	144	BakaG/Baka	West CAHG
Bakola	29	Bakola	West CAHG
Bakoya	20	Bakoya	West CAHG
Bedzan	24	Bedzan/Bezan	West CAHG
Aka	17	Biaka	West CAHG
Batwa East	35	Batwa	East CAHG
Efe & Sua	13	Mbuti	East CAHG
Bakiga	54	Bakiga	Non_HG
Bariba	20	Bariba	Non_HG
Fon	12	Fon	Non_HG
Nzebi	61	Nzebi	Non_HG
Yoruba	32	Yoruba	Non_HG

Table S5. Multiple matrix regressions assessing the relationship between geographical, genetic, ecological and cultural distance with masked dataset (CAHG genomic segments). Bold numbers represent statistically significant coefficients at a significance threshold of 0.05.

	**Musical instruments**		**Subsistence tools**	
	Coefficient	*p value*	Coefficient	*p value*
Genes (cont. Geography)	0.185	0.191	0.244	0.164
Genes (cont. ecology)	**0.414**	**0.018**	0.142	0.274
Geography (cont. Genes)	0.269	0.088	-0.031	0.578
Geography (cont. Ecology)	**0.395**	**0.008**	0.0457	0.373
Ecology (cont. Geography)	-0.029	0.559	**0.496**	**0.015**
Ecology (cont. Genes)	-0.033	0.541	0.309	0.119

Table S6. Results of the (a) overall and (b) a posteriori CADM tests, using geographical, ecological, musical instrument repertoire and genetic distance matrices using CAHG ancestry segments exclusively. (c) The complementary one-tailed rank tests are also shown for equivalent tests including genetic distance matrices built from each of the other three genetic datasets. P = permutational probability, P_H = permutational probability after Holm adjustment, r = Mantel statistic using ranks. All probabilities are based upon 1000 permutations. Bold numbers represent significant statistics at a significance threshold of 0.05.

**(a)**	**Overall CADM tests**			
	Kendall's *W*	0.594		
	Friedman's chi-squared	83.097	***P*<0.001**	
**(b)**	**A *posteriori* CADM tests**			
	Musical instruments	**P_H=0.014**		
	Geography	**P_H=0.008**		
	Ecology	**P_H=0.014**		
	Genes (CAHG ancestry)	**P_H=0.008**		
**(c)**	**One-tailed rank tests**			
		Musical instruments	Geography	Ecology
	Musical instruments			
	Geography	***r*=0.503** ***P*=0.008**		
	Ecology	*r*=0.250 *P*=0.118	***r*=0.343** ***P*=0.027**	
	Genes (CAHG ancestry)	***r*=0.416** ***P*=0.013**	***r*=0.743** ***P*=0.002**	***r*=0.494** ***P*=0.016**
	Genes (CAHG ancestry – IBD)	***r*=0.422** ***P*=0.014**	***r*=0.753** ***P*=0.001**	***r*=0.498** ***P*=0.011**
	Genes (full dataset)	*r*=0.312 *P*=0.074	***r*=0.767** ***P*=0.002**	*r*=0.351 *P*=0.052
	Genes (Bantu ancestry)	*r*=-0.015 *P*=0.508	*r*=-0.181 *P*=0.666	*r*=-0.347 *P*=0.846

Table S7. Results of the (a) overall and (b) a posteriori CADM tests, using geographical, ecological, subsistence tool repertoire and genetic distance matrices using CAHG ancestry segments exclusively. (c) The complementary one-tailed rank tests are also shown for equivalent tests including genetic distance matrices built from each of the other three genetic datasets. *P* = permutational probability, P_H = permutational probability after Holm adjustment, *r* = Mantel statistic using ranks. All probabilities are based upon 1000 permutations. Bold numbers represent significant statistics at a significance threshold of 0.05.

**(a)**	**Overall CADM tests**			
	Kendall's *W*	0.543		
	Friedman's chi-squared	76.088	***P*<0.001**	
**(b)**	**A *posteriori* CADM tests**			
	Subsistence tools	P_H=0.123		
	Geography	**P_H=0.009**		
	Ecology	**P_H=0.008**		
	Genes (CAHG ancestry)	**P_H=0. 012**		
**(c)**	**One-tailed rank tests**			
		Subsistence tools	Geography	Ecology
	Subsistence tools			
	Geography	*r*=0.207<br>*P*=0.107		
	Ecology	***r*=0.480**<br>***P*=0.023**	***r*=0.353**<br>***P*=0.027**	
	Genes (CAHG ancestry)	*r*=0.211<br>*P*=0.156	***r*=0.743**<br>***P*=0.002**	***r*=0.493**<br>***P*=0.008**
	Genes (CAHG ancestry – IBD)	*r*=0.215<br>*P*=0.160	***r*=0.753**<br>***P*=0.005**	***r*=0.499**<br>***P*=0.012**
	Genes (full dataset)	*r*=0.087<br>*P*=0.362	*r*=0.767<br>*P*=0.002	*r*=0.351<br>*P*=0.053
	Genes (Bantu ancestry)	*r*=-0.242<br>*P*=0.762	*r*=-0.180<br>*P*=0.660	*r*=-0.347<br>*P*=0.872

Table S8. Mantel tests predicting structure of genetic diversity (Fst) using CAHG ancestry components. *P* values are adjusted with Benjamini Hochberg procedure. Bold numbers represent significant statistics at a significance threshold of 0.05.

	Genetic diversity (F_{ST})	
	Statistic	*Adj p*
Geography	**0.818**	**0.001**
Ecology	**0.434**	**0.022**
Geography (cont. ecology)	**0.746**	**0.001**
Ecology (cont. geography)	0.201	0.182

Table S9. Estimated admixture proportion from hypothesised admixture source location in *Spacemix* (taken from MCMC run with highest posterior probability).

Population	Proportion of genome from inferred admixture source
Bedzan	0.128
Baka	0.110
Batwa (East)	0.063
Aka	0.061
Bakoya	0.054
Babongo	0.010
Bakola	0.002
Efe & Sua	0.002

Table S10. Geographical coordinates used for each of the CAHG populations (centroid of their range), as well as *SpaceMix* pseudo-coordinates based on genetics (using only CAHG components), musical instrument repertoires, and subsistence tool repertoires.

Group	Lon GEOG	Lat GEOG	Lon GEN	Lat GEN	Lon MUS	Lat MUS	Lon SUB	Lat SUB
Baka	14.14	2.35	12.59	-2.19	15.26	1.99	13.89	1.72
Bakoya	14.48	0.69	14.09	4.04	12.66	1.94	12.29	1.45
Bakola	10.15	3.01	14	4.21	11.08	2.58	14.2	1.63
Efe	29.33	1.81	29.29	1.76	29.66	2.19	29.76	2.55
Sua	28.38	1.62	29.29	1.76	28.7	1.56	27.63	3.91
Batwa	29.35	-2.37	22.26	-0.1	29.07	-2.18	27.31	-4.29
Aka	17.22	2.43	13.46	-0.09	16.94	0.87	14.85	2.13
Bedzan	11.36	5.87	14.6	2.61	11.59	3.71	13.28	7.36
Babongo	11.98	-2	16.77	1.37	11.45	0.52	12.93	-3.08

Table S11. Total number of shared IBD segments >1cM between individuals belonging to CAHG populations (only within CAHG genomic components)

	Babongo	Baka	Bakola	Bakoya	Batwa	Bedzan	Aka	Efe & Sua
Babongo	1385	2790	394	330	378	639	527	68
Baka	2790	71819	4549	4118	1714	12155	28922	339
Bakola	394	4549	981	835	390	1718	1325	32
Bakoya	330	4118	835	882	284	1623	1386	31
Batwa	378	1714	390	284	4245	393	213	100
Bedzan	639	12155	1718	1623	393	9904	4735	127
Aka	527	28922	1325	1386	213	4735	7358	77
Efe & Sua	68	339	32	31	100	127	77	3015

Table S12. Total length of IBD segments >1cM among shared all individuals from each CAHG population pair (only within CAHG genomic components)

	Babongo	Baka	Bakola	Bakoya	Batwa	Bedzan	Aka	Efe & Sua
Babongo	16893.098	12601.295	1968.582	1650.36	2423.201	1664.461	1114.199	128.321
Baka	12601.295	254052.515	15724.828	13071.769	8204.875	29727.654	78632.294	683.507
Bakola	1968.582	15724.828	4172.076	3090.363	2955.117	4285.595	2980.787	66.201
Bakoya	1650.36	13071.769	3090.363	3590.363	2328.025	3976.952	3209.948	57.697
Batwa	2423.201	8204.875	2955.117	2328.025	20928.371	1379.269	443.288	214.79
Bedzan	1664.461	29727.654	4285.595	3976.952	1379.269	64284.796	11427.79	247.443
Aka	1114.199	78632.294	2980.787	3209.948	443.288	11427.79	31292.914	149.019
Efe & Sua	128.321	683.507	66.201	57.697	214.79	247.443	149.019	9804.437

Table S13. Shared lexical items between CAHG groups. The "Variation in word" column indicates all variants in original terminology used to design that object that were lumped under the same word category. Y= Yes, N= No. *Denote word variations that are likely shared but evidence is not conclusive.

Word	Variation in word	Meaning	Populations sharing word and meaning	Object CAHG unique?	Word CAHG unique?
**Musical instruments**					
ndumu	ndùmù, ndumu	drum (conical)	Aka, Baka, Bakoya,	N	N
ngom	ngomo, ngoma, ŋgɔm, ngomu, ngom	drum (cylyndrical)	Bakoya, Bakola, Batwa (West), Babongo	N	N
hindehu	hìndèhú, èlehú	flute (small, simple)	Aka, Baka	Y	Y
ngombi	ngòmbi, ngombi, bo.ngombe, ngɔ̀ mbī	harp	Aka, Babongo, Baka, Batwa (West)	Y	Y
bogongo	bògóngó, bògongo	zither harp	Aka, Baka	Y	Y
ligbebe	lìgbégbé<br><br>lìgbebe, li.gbo:gbo, litchèké*	rattle (basket)	Aka, Baka, Efe, Babongo*	N	N
soko	sòkò, soko, soki, ma.soku, sàkà	Rattle	Aka, Babongo, Baka, Bakoya	N	N
baka	mbaka, bákà	struck beam (bamboo)	Babongo, Bakola	Y	Y
bàndá	mbàndá, banza, mbàndā	rhythmic sticks (hunting ritual)	Aka, Baka, Sua	Y	Y
mobio	mòbìó, mobio	flute (notched)	Aka, Baka	Y	Y
ruma	ruma, baruma	flute	Efe, Sua	N	Y
sanze	sànze, sanzi, sanza	lamellaphone	Aka, Babongo	N	N
ashaka	ashaka	ankle rattles (made with seeds and tied with a string)	Aka, Baka	Y	Y
kúbù	kúbù	high-pitched drum (played alongside mokinda)	Aka, Baka	Y	Y
mokinda	mòkíndá, mokinda	single-skinned drum	Aka, Baka	Y	Y
kembe	likembe, ikembe, kembe, kembi	lamellaphone	Baka, Batwa (East), Efe, Sua	N	N

mbɛ	mbɛ, mgbæ	drum	Bedzan, Bakola	N	N
ngbídí	èngbítí, língbidi, engbídí, kittingbi	musical bow (2 strings, played by females)	Aka, Baka, Efe	Y	Y
gongo	mugongo, mgongo, ngàngo*	musical bow (1 string)	Babongo, Baka*	Y	Y
ngindi	ngindi, ngadi*	musical bow	Aka, Bakoya*	Y	Y
beka	di.beka, di.boka, mo.beke, móbéké	whistle (papaya leafstalk)	Aka, Babongo	Y	Y
pole.pole	pole.pole, polo, polo	whistle (seeds)	Aka, Baka	Y	Y
bisa	besa, bisa, mangisa*	rattle	Babongo, Baka*	N	Y
**Subsistence tools**					
apekulo	apeku.lo, apokolo	cable snare	Baka, Bakoya	N	Y
api	api, apí, abi	arrow	Efe, Sua	N	N
sàwàla	sàwàla	leather sac	Aka, Baka	Y	Y
mokobe	mòkòbé, mokobe	honey container	Aka, Baka	Y	Y
mbánji	mbánji, mbadi	wooden tip arrow	Baka, Batwa (West)	Y	Y
bànzà	mbànzà, di-banzika	metal tip arrow	Aka, Bakoya	N	N
ikua	di.kuya, íkùa	basket	Aka, Bakoya	N	Y
nbgango	ngbángò, bo.ngango	bow (hunting)	Aka, Batwa* (West)	N	N
pèndi	pèndi	container	Aka, Baka	N	N
konga	e.kòngá, ekonga, kongo, dikono*	spear	Aka, Bakoya*, Sua	N	N
leki	éléki, lékè, mò.lékè	trap	Aka, Sua	N	Y

Table S14. Coefficients of Zero-Inflated Poisson model predicting the sharing of musical instrument words between CAHG populations as a function of genetic, and linguistic distance calculated with PMI scores (top) and geographic, and linguistic distance calculated with PMI scores (bottom), using the full linguistic dataset. All predictors were standardized. Bold numbers represent significant coefficients at a significance threshold of 0.05.

	**Estimate**	**Std. Error**	**z value**	***p* value**
Count model coefficients				
Intercept	0.423	0.274	1.543	0.123
Genetic distance (CAHG ancestry)	**-0.769**	**0.233**	**-3.299**	**0.001**
Linguistic distance (PMI score)	0.360	0.191	1.882	0.060
Zero-inflation model coefficients				
Intercept	-0.518	0.630	-0.821	0.411
Genetic distance (CAHG ancestry)	0.758	0.593	1.279	0.201
Linguistic distance (PMI score)	1.201	0.710	1.691	0.091

	**Estimate**	**Std. Error**	**z value**	***p* value**
Count model coefficients				
Intercept	0.454	0.276	1.646	0.100
Geographical distance	-0.451	0.263	-1.715	0.086
Linguistic distance (PMI score)	0.080	0.155	0.518	0.604
Zero-inflation model coefficients				
Intercept	-0.407	0.554	-0.734	0.463
Geographical distance	**1.296**	**0.545**	**2.378**	**0.017**
Linguistic distance (PMI score)	0.431	0.467	0.923	0.356

Table S15. Coefficients of Zero-Inflated Poisson model predicting the sharing of musical instrument words between CAHG populations as a function of genetic, and linguistic distance calculated using Glottolog (top) and geographic, and linguistic distance calculated using Glottolog (bottom), using the full linguistic dataset. All predictors were standardized. Bold numbers represent significant coefficients at a significance threshold of 0.05.

	**Estimate**	**Std. Error**	**z value**	***p* value**
Count model coefficients				
Intercept	0.298	0.313	0.954	0.340
Genetic distance (CAHG ancestry)	**-0.793**	**0.254**	**-3.124**	**0.002**
Linguistic distance (Glottolog)	0.279	0.154	1.810	0.070
Zero-inflation model coefficients				
Intercept	-0.523	0.663	-0.789	0.430
Genetic distance (CAHG ancestry)	0.628	0.623	1.007	0.314
Linguistic distance (Glottolog)	0.186	0.523	0.356	0.722

	**Estimate**	**Std. Error**	**z value**	***p* value**
Count model coefficients				
Intercept	0.453	0.258	1.757	0.079
Geographical distance	-0.444	0.241	-1.847	0.065
Linguistic distance (Glottolog)	0.255	0.153	1.668	0.095
Zero-inflation model coefficients				
Intercept	-0.397	0.523	-0.760	0.447
Geographical distance	**1.244**	**0.529**	**2.353**	**0.019**
Linguistic distance (Glottolog)	0.205	0.459	0.447	0.655

Table S16. Coefficients of Zero-Inflated Poisson model predicting the sharing of musical instrument words between CAHG populations as a function of genetic, and linguistic distance calculated using Koile et al.'s phylogeny (top) and geographic, and linguistic distance calculated using Koile et al.'s phylogeny (bottom), using the full linguistic dataset. All predictors were standardized. Bold numbers represent significant coefficients at a significance threshold of 0.05.

	**Estimate**	**Std. Error**	**z value**	***p* value**
Count model coefficients				
Intercept	0.004	0.255	0.014	0.989
Genetic distance (CAHG ancestry)	**-0.978**	**0.208**	**-4.700**	**0.000**
Linguistic distance (phylogenetic)	**0.570**	**0.169**	**3.365**	**0.001**
Zero-inflation model coefficients				
Intercept	-6.072	73.581	-0.083	0.934
Genetic distance (CAHG ancestry)	0.208	0.645	0.322	0.747
Linguistic distance (phylogenetic)	6.631	88.745	0.075	0.940

	**Estimate**	**Std. Error**	**z value**	***p* value**
Count model coefficients				
Intercept	0.366	0.298	1.231	0.218
Geographical distance	-0.516	0.275	-1.879	0.060
Linguistic distance (phylogenetic)	**0.367**	**0.186**	**1.969**	**0.049**
Zero-inflation model coefficients				
Intercept	-0.614	0.832	-0.738	0.460
Geographical distance	1.087	0.618	1.757	0.079
Linguistic distance (phylogenetic)	0.562	1.054	0.533	0.594

Table S17. Coefficients of Zero-Inflated Poisson model predicting the sharing of musical instrument words between CAHG populations as a function of genetic, and linguistic distance calculated with PMI scores (top) and geographic, and linguistic distance calculated with PMI scores (bottom), using the subset of words that were both unique to CAHG and clear cases of sharing. All predictors were standardized. Bold numbers represent significant coefficients at a significance threshold of 0.05.

	**Estimate**	**Std. Error**	**z value**	***p* value**
Count model coefficients				
Intercept	-0.895	0.603	-1.485	0.137
Genetic distance (CAHG ancestry)	**-1.621**	**0.434**	**-3.731**	**<0.001**
Linguistic distance (PMI score)	-1.528	0.318	-0.153	0.664
Zero-inflation model coefficients				
Intercept	-0.134	1.156	-0.116	0.908
Genetic distance (CAHG ancestry)	0.342	0.867	0.394	0.693
Linguistic distance (PMI score)	0.082	1.081	0.076	0.940

	**Estimate**	**Std. Error**	**z value**	***p* value**
Count model coefficients				
Intercept	-0.461	0.394	-1.171	0.242
Geographical distance	**-1.281**	**0.344**	**-3.722**	**<0.001**
Linguistic distance (PMI score)	0.080	0.155	0.518	0.604
Zero-inflation model coefficients				
Intercept	-0.737	1.279	-0.577	0.564
Geographical distance	-0.838	1.256	-0.667	0.505
Linguistic distance (PMI score)	-0.479	0.547	-0.875	0.381

Table S18. Coefficients of Zero-Inflated Poisson model predicting the sharing of musical instrument words between CAHG populations as a function of genetic, and linguistic distance calculated using Glottolog (top) and geographic, and linguistic distance calculated using Glottolog (bottom), using the subset of words that were both unique to CAHG and clear cases of sharing. All predictors were standardized. Bold numbers represent significant coefficients at a significance threshold of 0.05.

	**Estimate**	**Std. Error**	**z value**	***p* value**
Count model coefficients				
Intercept	-0.699	0.753	-0.928	0.353
Genetic distance (CAHG ancestry)	**-1.770**	**0.548**	**-3.272**	**0.001**
Linguistic distance (Glottolog)	0.005	0.294	0.018	0.986
Zero-inflation model coefficients				
Intercept	-0.341	1.506	-0.226	0.821
Genetic distance (CAHG ancestry)	0.226	1.005	0.225	0.822
Linguistic distance (Glottolog)	-0.300	0.554	-0.541	0.588

	**Estimate**	**Std. Error**	**z value**	***p* value**
Count model coefficients				
Intercept	-0.401	0.451	-0.889	0.374
Geographical distance	**-1.417**	**0.345**	**-4.108**	**<0.001**
Linguistic distance (Glottolog)	0.187	0.230	0.813	0.416
Zero-inflation model coefficients				
Intercept	-0.504	1.316	-0.383	0.702
Geographical distance	-0.587	1.324	-0.444	0.657
Linguistic distance (Glottolog)	0.228	0.505	0.451	0.652

Table S19. Coefficients of Zero-Inflated Poisson model predicting the sharing of musical instrument words between CAHG populations as a function of genetic, and linguistic distance calculated using Koile et al.'s phylogeny (top) and geographic, and linguistic distance calculated using Koile et al.'s phylogeny (bottom), using the subset of words that were both unique to CAHG and clear cases of sharing. All predictors were standardized. Bold numbers represent significant coefficients at a significance threshold of 0.05.

	**Estimate**	**Std. Error**	**z value**	***p* value**
Count model coefficients				
Intercept	-0.896	0.502	-1.786	0.074
Genetic distance (CAHG ancestry)	**-1.224**	**0.370**	**-3.312**	**<0.001**
Linguistic distance (phylogenetic)	-0.002	0.412	-0.007	0.994
Zero-inflation model coefficients				
Intercept	-6.436	97.788	-0.066	0.948
Genetic distance (CAHG ancestry)	0.393	0.814	0.483	0.629
Linguistic distance (phylogenetic)	7.817	117.949	0.066	0.947

	**Estimate**	**Std. Error**	**z value**	***p* value**
Count model coefficients				
Intercept	-0.562	0.358	-1.571	0.116
Geographical distance	**-1.011**	**0.346**	**-2.922**	**0.003**
Linguistic distance (phylogenetic)	**0.751**	**0.295**	**2.547**	**0.011**
Zero-inflation model coefficients				
Intercept	-1.333	1.522	-0.876	0.381
Geographical distance	-0.635	1.138	-0.558	0.577
Linguistic distance (phylogenetic)	1.431	1.629	0.878	0.380

Table S20. Number of iterations used for each K value in ADMIXTURE analyses

K value	Iterations
2	58
3	53
4	50
5	46
6	55
7	61
8	50

Table S21. Mean proportion of ancestry components from out ADMIXTURE run at K=3. CAHG_1 and CAHG_2 refer to ancestry components associated with Western and Eastern CAHG respectively. AGR refer to ancestry components associated with African agriculturalist populations.

**Population**	**CAHG_1**	**CAHG_2**	**AGR**
Babongo	0.378	0.100	0.523
Baka	0.851	0.012	0.137
Bakola	0.663	0.109	0.228
Bakoya	0.656	0.110	0.234
Batwa	0.014	0.768	0.219
Bedzan	0.522	0.078	0.400
Aka	0.763	0.080	0.157
Efe & Sua	0.225	0.775	0.000001

Table S22. Confusion matrix from *Gnomix* analyses indicating the number and proportion of correctly classified haplotypes of CAHG ancestry (CAHG_corr) and of agriculturalist associated ancestry (AGR_corr) in the validation dataset per chromosome.

CHR	CAHG_corr	CAHG_miss	CAHG_corr_prop	AGR_corr	AGR_miss	AGR_corr_prop
1	58444	9551	0.86	51978	9537	0.84
2	52461	7014	0.88	56820	8355	0.87
3	50155	7455	0.87	38596	7924	0.83
4	44889	5078	0.90	40522	8241	0.83
5	40876	7785	0.84	37714	7765	0.83
6	33410	7876	0.81	41693	5941	0.88
7	38056	6696	0.85	32656	6922	0.83
8	32656	3521	0.90	33440	8059	0.81
9	35558	6216	0.85	35518	5328	0.87
10	36937	3743	0.91	32678	9172	0.78
11	31328	3983	0.89	31732	5767	0.85
12	37802	2136	0.95	32713	6549	0.83
13	22428	4587	0.83	27022	3833	0.88
14	25708	1931	0.93	21842	3889	0.85
15	29849	3396	0.90	27538	6267	0.81
16	26601	3943	0.87	25174	4582	0.85
17	25540	4653	0.85	23535	4232	0.85
18	27416	2208	0.93	18989	6017	0.76
19	20253	1700	0.92	20436	5221	0.80
20	22525	3576	0.86	21440	2949	0.88
21	10831	2108	0.84	12664	2108	0.86
22	14376	1117	0.93	15566	3231	0.83

Dataset S1. (separate file)
All material cultural objects included in this study.

Dataset S2. (separate file)
Vocabulary items available, list of cognate sets, additional notes on linguistic terminology, criteria used for selecting cognate sets and additional references related to linguistic analyses.
