# Supplementary material for: Deep history of cultural and linguistic evolution among Central African hunter-gatherers
Source: Nat Hum Behav. 2024 May 27;8(7):1263–75. doi: 10.1038/s41562-024-01891-y (PMC11272592; doi:10.1038/s41562-024-01891-y)
Supplement: Supplementary file 2 — Reporting Summary [file 41562_2024_1891_MOESM2_ESM.pdf]

Corresponding author(s): Cecilia Padilla-Iglesias
Andrea Migliano

Last updated by author(s): 29.03.2024

Reporting Summary

Nature Portfolio wishes to improve the reproducibility of the work that we publish. This form provides structure for consistency and transparency in reporting. For further information on Nature Portfolio policies, see our Editorial Policies and the Editorial Policy Checklist.

Statistics

For all statistical analyses, confirm that the following items are present in the figure legend, table legend, main text, or Methods section.

n/a	Confirmed	
☐	☒	The exact sample size (n) for each experimental group/condition, given as a discrete number and unit of measurement
☐	☒	A statement on whether measurements were taken from distinct samples or whether the same sample was measured repeatedly
☐	☒	The statistical test(s) used AND whether they are one- or two-sided<br>*Only common tests should be described solely by name; describe more complex techniques in the Methods section.*
☐	☒	A description of all covariates tested
☐	☒	A description of any assumptions or corrections, such as tests of normality and adjustment for multiple comparisons
☐	☒	A full description of the statistical parameters including central tendency (e.g. means) or other basic estimates (e.g. regression coefficient) AND variation (e.g. standard deviation) or associated estimates of uncertainty (e.g. confidence intervals)
☐	☒	For null hypothesis testing, the test statistic (e.g. F, t, r) with confidence intervals, effect sizes, degrees of freedom and P value noted<br>*Give P values as exact values whenever suitable.*
☒	☐	For Bayesian analysis, information on the choice of priors and Markov chain Monte Carlo settings
☒	☐	For hierarchical and complex designs, identification of the appropriate level for tests and full reporting of outcomes
☒	☐	Estimates of effect sizes (e.g. Cohen's d, Pearson's r), indicating how they were calculated

Our web collection on statistics for biologists contains articles on many of the points above.

Software and code

Policy information about availability of computer code

Data collection

Provide a description of all commercial, open source and custom code used to collect the data in this study, specifying the version used OR state that no software was used.

Data analysis

All data and code required to reproduce the results in the manuscript and supplementary information is available in the following GitHub repository: https://github.com/ceciliapad/GenCultEvo. We used the following software:

- Gnomix (https://github.com/AI-sandbox/gnomix)
- PLINK (PLINK v1.90b6.12)
- R (R version 4.3.1)
- KING (https://www.kingrelatedness.com)
- Spacemix (https://github.com/gbradburd/SpaceMix)
- ADMIXTURE (ADMIXTURE/1.3.0)
- Beagle 5.3
- RefinedIBD(https://faculty.washington.edu/browning/refined-ibd.html)
- ape R package (version 5.7)
- phangorn R package (version 2.10)

The remaining packages are specified not in the methods but in the scripts included in the Github repository.

For manuscripts utilizing custom algorithms or software that are central to the research but not yet described in published literature, software must be made available to editors and reviewers. We strongly encourage code deposition in a community repository (e.g. GitHub). See the Nature Portfolio guidelines for submitting code & software for further information.

Data

Policy information about availability of data

All manuscripts must include a data availability statement. This statement should provide the following information, where applicable:

- Accession codes, unique identifiers, or web links for publicly available datasets
- A description of any restrictions on data availability
- For clinical datasets or third party data, please ensure that the statement adheres to our policy

Processed genetic data, and raw cultural and linguistic data required to reproduce all the results reported in the manuscript and supplementary are available in the following Github repository: https://github.com/ceciliapad/GenCultEvo. Raw genome-wide SNP data is available in public servers EGA (Accession number EGAS00001002078 and EGAC00001000139) and dbGaP (Accession numbers phs000449.v2.p1 and phs001780.v1.p1).
Language phylogenies were obtained from the ASJP database (https://asjp.clld.org), Koile et al. (2022) and Glottolog v.4.8 (https://glottolog.org)

Research involving human participants, their data, or biological material

Policy information about studies with human participants or human data. See also policy information about sex, gender (identity/presentation), and sexual orientation and race, ethnicity and racism.

Reporting on sex and gender	No subjects were involved.
Reporting on race, ethnicity, or other socially relevant groupings	No subjects were involved but cultural information on Central African hunter-gatherer populations were obtained from museum collections, ethnographers and primary literature. Population named reflect their recognised ethnicities and primary literature.
Population characteristics	We compiled published cultural, genetic and location data from each population and museum data on cultural artifacts.
Recruitment	No subjects
Ethics oversight	Research did not require human subjects or animals and no ethics oversight was required, but a data access committee gave ethics approval for our use of the above-mentioned genetic datasets for the purpose of this project.

Note that full information on the approval of the study protocol must also be provided in the manuscript.

Field-specific reporting

Please select the one below that is the best fit for your research. If you are not sure, read the appropriate sections before making your selection.

☐ Life sciences ☐ Behavioural & social sciences ☒ Ecological, evolutionary & environmental sciences

For a reference copy of the document with all sections, see nature.com/documents/nr-reporting-summary-flat.pdf

Ecological, evolutionary & environmental sciences study design

All studies must disclose on these points even when the disclosure is negative.

Study description	This study presents an analysis of cultural, genetic and linguistic data from Central African hunter-gatherer populations.
Research sample	Published data on words, genetic sequences and museum data on cultural artifacts from Central African hunter-gatherer populations.
Sampling strategy	We collected all data available on cultural artifacts and vocabulary items.
Data collection	Primary literature and museum visits
Timing and spatial scale	A few months of visits to libraries and museums. Spatial scale is the entire Congo Basin.
Data exclusions	No exclusion
Reproducibility	All data and code are available
Randomization	Not applicable
Blinding	Not applicable

Did the study involve field work? ☐ Yes ☒ No

Reporting for specific materials, systems and methods

We require information from authors about some types of materials, experimental systems and methods used in many studies. Here, indicate whether each material, system or method listed is relevant to your study. If you are not sure if a list item applies to your research, read the appropriate section before selecting a response.

Materials & experimental systems

n/a	Involved in the study
☒	☐ Antibodies
☒	☐ Eukaryotic cell lines
☒	☐ Palaeontology and archaeology
☒	☐ Animals and other organisms
☒	☐ Clinical data
☒	☐ Dual use research of concern
☒	☐ Plants

Methods

n/a	Involved in the study
☒	☐ ChIP-seq
☒	☐ Flow cytometry
☒	☐ MRI-based neuroimaging

Plants

Seed stocks

Report on the source of all seed stocks or other plant material used. If applicable, state the seed stock centre and catalogue number. If plant specimens were collected from the field, describe the collection location, date and sampling procedures.

Novel plant genotypes

Describe the methods by which all novel plant genotypes were produced. This includes those generated by transgenic approaches, gene editing, chemical/radiation-based mutagenesis and hybridization. For transgenic lines, describe the transformation method, the number of independent lines analyzed and the generation upon which experiments were performed. For gene-edited lines, describe the editor used, the endogenous sequence targeted for editing, the targeting guide RNA sequence (if applicable) and how the editor was applied.

Authentication

Describe any authentication procedures for each seed stock used or novel genotype generated. Describe any experiments used to assess the effect of a mutation and, where applicable, how potential secondary effects (e.g. second site T-DNA insertions, mosiacism, off-target gene editing) were examined.
